# Adjusted Age-Adjusted Charlson Comorbidity Index Score as a Risk Measure of Perioperative Mortality before Cancer Surgery

**DOI:** 10.1371/journal.pone.0148076

**Published:** 2016-02-05

**Authors:** Chun-Ming Chang, Wen-Yao Yin, Chang-Kao Wei, Chin-Chia Wu, Yu-Chieh Su, Chia-Hui Yu, Ching-Chih Lee

**Affiliations:** 1 Department of Surgery, Dalin Tzu Chi Hospital, Buddhist Tzu Chi Medical Foundation, Chiayi, Taiwan; 2 Department of Otorhinolaryngology, Head and Neck Surgery, Kaohsiung Veterans General Hospital, Kaohsiung, Taiwan; 3 School of Medicine, National Defense Medical Center, Taipei, Taiwan; 4 Division of Hematology-Oncology, Department of Internal Medicine, Dalin Tzu Chi Hospital, Buddhist Tzu Chi Medical Foundation, Chiayi, Taiwan; 5 Cancer Center, Dalin Tzu Chi Hospital, Buddhist Tzu Chi Medical Foundation, Chiayi, Taiwan; 6 School of Medicine, Tzu Chi University, Hualian, Taiwan; 7 Department of Otolaryngology, Head and Neck Surgery, Tri-Service General Hospital, Taipei, Taiwan; University of Kentucky, UNITED STATES

## Abstract

**Background:**

Identification of patients at risk of death from cancer surgery should aid in preoperative preparation. The purpose of this study is to assess and adjust the age-adjusted Charlson comorbidity index (ACCI) to identify cancer patients with increased risk of perioperative mortality.

**Methods:**

We identified 156,151 patients undergoing surgery for one of the ten common cancers between 2007 and 2011 in the Taiwan National Health Insurance Research Database. Half of the patients were randomly selected, and a multivariate logistic regression analysis was used to develop an adjusted-ACCI score for estimating the risk of 90-day mortality by variables from the original ACCI. The score was validated. The association between the score and perioperative mortality was analyzed.

**Results:**

The adjusted-ACCI score yield a better discrimination on mortality after cancer surgery than the original ACCI score, with c-statics of 0.75 versus 0.71. Over 80 years of age, 70–80 years, and renal disease had the strongest impact on mortality, hazard ratios 8.40, 3.63, and 3.09 (*P* < 0.001), respectively. The overall 90-day mortality rates in the entire cohort varied from 0.9%, 2.9%, 7.0%, and 13.2% in four risk groups stratifying by the adjusted-ACCI score; the adjusted hazard ratio for score 4–7, 8–11, and ≥ 12 was 2.84, 6.07, and 11.17 (*P* < 0.001), respectively, in 90-day mortality compared to score 0–3.

**Conclusions:**

The adjusted-ACCI score helps to identify patients with a higher risk of 90-day mortality after cancer surgery. It might be particularly helpful for preoperative evaluation of patients over 80 years of age.

## Introduction

The numbers of cancer patients are increasing yearly. There were 14.1 million new cancer cases in 2012 worldwide [1]. Cancer is a leading cause of death, and it accounted for 8.2 million cancer deaths in 2012 worldwide.[1] With advances in healthcare and cancer treatment, the combined cancer death rate has been continually declining for decades in Taiwan as well as in other developed countries [2,3]. For cancer patients who undergo surgical treatment, their safety is of utmost concern. Although difficult operations have become standardized (such as esophageal, pancreatic and hepatic operations), and total perioperative mortality has decreased over time [4], death is still one of the most feared complications of surgery. While the number of cancer patients remains high worldwide, and baseline risk status of patients who presented for surgery increased over the decades [4], the magnitudes of risk of death related to various cancer operations are not well understood. Preoperative stratification of at-risk cancer patients is needed to improve preoperative evaluation and preparation.

The Charlson comorbidity index is one of the measures for health status, which has been used to evaluate the outcomes in many clinical settings. Compromised organ function affects the therapeutic planning and outcome of treatment. Previous studies on various benign diseases and malignancy showed significantly lower disease-specific outcomes and poorer short-term and long-term survivals for patients with a higher burden of comorbidity [5–20]. Additionally, age was determined to be a significant factor for survival and was subsequently incorporated into the Charlson comorbidity score to create a single index accounting for both age and comorbidity, ie, the age-adjusted Charlson comorbidity index (ACCI) [21]. The number of comorbidities was suggested to increase with aging [22]. Some studies have used it to predict certain disease-specific outcomes [23–25]. However, the ACCI was developed about two decades ago, with the advancement in medical care, it is not clear whether the weight of each parameter in the ACCI remains the same when applying it for measuring perioperative risks for recent cancer operations.

In addition to the use of clinical data for outcome and risk measures as Chalson first intended, these clinical comorbidities have been translated into the ICD code and for use as a risk-adjustment tool using the administrative data [6,8–10,15,16,18–20,26]. Therefore, in this study we used the ICD-coded data to support clinical care in an attempt to make a simple and easy to use score for cancer surgery preoperative risk evaluation. We used a population-based national database of Taiwanese patients with common cancers who underwent cancer surgery between 2007 and 2011 for this study. This study had three purposes: first, taking the original ACCI score as a risk measure for 90-day mortality after cancer surgery and using our patients’ data to validate it, and then developing and validating an adjusted-ACCI score based on our patients’ data in order to yield a better discrimination than the original ACCI score for predicting 90-day mortality after cancer surgery. Second, identifying the preoperative risk factors associated with the 90-day mortality risk after cancer surgery. Third, using the adjusted-ACCI score as a risk measure to determine its association with 90-day mortality after cancer surgery.

## Materials and Mathods

### Ethics statements

This study was initiated after receiving approval from the Institutional Review Board of the Buddhist Dalin Tzu Chi General Hospital, Taiwan. The identification numbers and personal information of any individual patients were not included in the secondary files; therefore, the review board stated that written patient consent was not required in this study.

### Patients and study design

This study used data from 2007 to 2011 from the Taiwan National Health Insurance Research Database (NHIRD, which covers medical benefit claims for over 23 million people (approximately 99% of Taiwan’s population) [27]. Taiwan’s national health insurance system provides universal insurance coverage and comprehensive services. It is a single-payer system with the government as sole insurer. The database was monitored for completeness and accuracy by Taiwan’s Department of Health. Patients who underwent a surgical procedure for one of the ten common cancer types leading to death in Taiwan [2] (lung and bronchus, liver and intrahepatic bile duct, colorectum, breast, oral cavity and pharynx, stomach, prostate, pancreas, esophagus, and cervix) between 2007 and 2011 were included. The surgical procedures for each cancer were identified by their procedure codes. Cancer patients who underwent the related surgical procedures were enrolled. For patients with more than one type of cancer or who received more than one cancer operation for a recurrent disease during the five-year period of the study, only the first captured cancer code with the related surgical procedure was counted. A total of 418,617 patients with these cancers were identified from the NHIRD; among them, 163,536 patients received corresponding surgical procedures. We excluded 7385 patients with missing measurement data. The remaining 156,151 patients who underwent corresponding surgical procedures were enrolled. Patient deaths were identified from the National Register of Deaths Database.

### Measurements

The primary outcome measure was 90-day mortality, defined as death from all causes occurring after cancer surgery. The predictor variables were patient age, gender, cancer type, and pre-existing comorbidity. The original ACCI scores were calculated by use of the method reported by Charlson [21]. This index is a weighted measure that incorporates age and 19 different medical categories; each is weighted according to its impact on mortality (Table 1). The age is adjusted by calculating each decade after 40 years of age as one point in the original ACCI. For each decade after 40 years of age, a point is added until 4 points (1 point for age 41–50, 2 points for age 51–60, 3 points for age 61–70, 4 points for 71 years of age or older). The final score was calculated for each patient by taking into account all comorbid conditions present. We identified the presence or absence of pre-existing comorbidities in each patient by querying the Taiwan NHI database, using the International Classification of Diseases, Ninth Revision (ICD-9) codes. Any preoperative existing comorbidity was identified from the ICD-9 codes for inpatients within six months before surgery.

**Table 1:** Comorbidity distribution based on the age-adjusted Charlson comorbidities, *n* = 156,151.

Variable	Point	Number of patients (%)
Age		
40≧Age	0	11317(7.2)
50≧Age>40	1	30094(19.3)
60≧Age>50	2	41781(26.8)
70≧Age>60	3	33897(21.7)
80≧Age>70	4	27706(17.7)
Age>80	4	11356(7.3)
Myocardial infarction	1	305(0.2)
Congestive heart failure	1	966(0.6)
Peripheral vascular disease	1	167(0.1)
Cerebrovascular disease	1	1726(1.1)
Dementia	1	193(0.1)
Chronic pulmonary disease	1	3143(2.0)
Rheumatic disease	1	220(0.1)
Peptic ulcer disease	1	6414(4.1)
Mild liver disease	1	5184(3.3)
Diabetes mellitus without end-organ damage	1	7775(5.0)
Diabetes mellitus with end-organ damage	2	732(0.5)
Hemiplegia	2	118(0.1)
Renal disease	2	1053(0.7)
Any malignancy*	2	10737(6.9)
Lymphoma	2	267(0.2)
Leukemia	2	95(0.1)
Moderate liver disease	3	846(0.5)
Metastatic solid tumor	6	6135(3.9)
Acquired immunodeficiency syndrome (AIDS)	6	2(0.0)

*Patients with more than one type of cancer in this study population.

Renal disease: chronic glomerulonephritis; nephritis and nephropathy; chronic renal failure.

Mild liver disease: chronic hepatitis; alcoholic cirrhosis; biliary cirrhosis. Moderate liver disease: liver diseases with cirrhosis-related complications.

For outpatients, diseases that were coded three times or more in the records within six months before surgery were also identified from the ICD-9 codes. Metastatic disease might have been suspected before surgery, but in many cases it could not be proved without tissue examination. In those cases, many would not be coded in the records before surgery. To avoid underestimating the number of metastatic patients, instead of querying the metastatic status within six months before surgery, status was identified by ICD-9 codes 196.xx to 199.xx queried from patient discharges.

### Statistical analysis

The SPSS (version 15, SPSS Inc., Chicago, IL) was used for data analysis. A p-value of *P*<0.05 was used to determine statistical significance. To validate the original ACCI model in predicting 90-day mortality in cancer surgery, the risk score was calculated for each patient, and the discrimination was assessed using the area under receiver operating characteristic (ROC) curve. The result showed that the original ACCI model had an area under the ROC curve of 0.71 for predicting 90-day mortality in our patients undergoing cancer surgery.

Half of our study population was randomly selected (derivation population) for developing an adjusted-ACCI model, and the remaining half (validation population) was used to validate it. Univariate analyses were performed for the predictive variables from the original ACCI model. Those with statistical significance at the level of *P* < 0.05 were entered into a logistic regression model for hazard ratios in regard to 90-day mortality; multivariate logistic regression analysis was used to calculate the risk of mortality after adjustment for variables. The beta coefficients from the logistic regression model were used to yield an integer-based weighted point system for stratifying the 90-day mortality risks. The smallest coefficient was assigned a value of one point and the coefficients for the others were adjusted proportionally, rounding to the nearest integer. Individual scores were calculated by summing up the individual risk factor points.

Discrimination of the adjusted-ACCI model was assessed by using the area under the ROC curve within the derivation population set. For validating the adjusted-ACCI model, it was applied to the validation population, and its discrimination was assessed by ROC curve analysis. The results showed that the adjusted-ACCI model had an area under the ROC curve of 0.75. When applying it to the validation population set, the adjusted-ACCI model continued to discriminate well, with an area under the ROC curve of 0.75.

The next step was to stratify the risk scores into four risk groups: (1) score of 0 to 3; (2) score of 4 to 7; (3) score of 8 to 11 and (4) score of 12 and above. Then, the Cox proportional hazard regression model was used to assess the impact of adjusted-ACCI score on perioperative mortality after adjusting for patient demographic variables.

## Results

A total of 156,151 patients with various cancer diagnoses, who underwent corresponding surgical procedures between 2007 and 2011 were identified from the NHIRD. The age and comorbidities of patients are summarized in Table 1. Among them, 25 percent aged more than 70 years.

In the derivation population set (78,076 patients) for the adjusted-ACCI model, the hazard ration (HR) for patients in the 40–50 year old age group were not statistically different from those for patients younger than 40. Therefore, those aged under 50 were assigned as the referent in the adjusted-ACCI model. Table 2 shows the results of the multivariate logistic regression analysis of the adjusted-ACCI model with the predictors and risk score from the 78,076 patients randomly selected for the development of the model. After adjusting for other factors, within those parameters, patients over 80 years of age showed the strongest association with 90-day mortality risk (adjusted HR, 8.40; 95% confidence interval (CI), 7.16–9.86; *P* < 0.001), followed by patients aged 70–80, (adjusted HR, 3.63; 95% CI, 3.11–4.23; *P* < 0.001), and renal disease (chronic renal failure, glomerulonephritis, and nephrotic syndrome) (adjusted HR, 3.09; 95% CI, 2.34–4.10; *P* < 0.001).

**Table 2:** Development of the predictor score based on multivariate logistic regression analysis of the derivation set, *n* = 78,076.

	Coefficient β	Adjusted HR	95% CI	*P*-value	Risk score
50≧Age		1			
60≧Age>50	0.293	1.34	1.13–1.58	0.001	1
70≧Age>60	0.635	1.88	1.59–2.22	<0.001	3
80≧Age>70	1.289	3.63	3.11–4.23	<0.001	5
Age>80	2.129	8.40	7.16–9.86	<0.001	9
Congestive heart failure	0.722	2.05	1.53–2.76	<0.001	3
Dementia	0.763	2.14	1.21–3.78	0.008	3
Chronic pulmonary disease	0.319	1.37	1.11–1.69	0.003	1
Peptic Ulcer Disease	0.555	1.74	1.49–2.03	<0.001	2
Diabetes Mellitus	0.319	1.37	1.18–1.60	<0.001	1
Renal disease	1.131	3.09	2.34–4.10	<0.001	5
Lymphoma	1.000	2.71	1.43–5.13	0.002	4
Any malignancy	0.245	1.27	1.10–1.47	0.001	1
Metastatic solid tumor	0.918	2.50	2.28–2.74	<0.001	4
Mild Liver Disease	0.754	2.12	1.73–2.59	<0.001	3
Moderate Liver Disease	1.018	2.76	1.93–3.95	<0.001	4

Abbreviation: Adjusted HR, Adjusted hazard ratio; 95%CI, 95% confidence interval.

The adjusted-ACCI model discriminated both the derivation and validation set well, with an area under the ROC curve of 0.75 and 0.75, respectively. It yielded better discrimination for the 90-day mortality risk after cancer surgery than the original ACCI model (ROC curve of 0.71).

Table 3 shows the average adjusted-AACI score for those who live beyond 90 days and for those who died prior to 90 day. In all patients the score was 3.8±3.3 and 7.2±3.8 respectively, *P*<0.001. The average score of each type of cancer patients varied from 1.8 to 5.5 for those who live beyond 90 days and from 4.5 to 8.7 for those who died prior to 90 days.

**Table 3:** The average adjusted-AACI score for those who live beyond 90 days and for those who died prior to 90 days, Mean±SD, *n* = 156,151.

	Survival	Mortality	*P*-value
Total cancer patients	3.8±3.3	7.2±3.8	<0.001
Lung and bronchus	4.0±2.9	6.6±3.0	<0.001
Liver and intrahepatic bile duct	3.9±3.1	5.5±3.6	<0.001
Colorectum	5.1±3.4	8.4±3.6	<0.001
Breast	2.4±2.6	5.7±3.4	<0.001
Oral cavity and pharynx	2.6±2.6	5.0±3.5	<0.001
Stomach	5.5±3.6	8.2±3.6	<0.001
Prostate	5.2±3.0	8.7±3.5	<0.001
Pancreas	4.9±3.2	7.0±3.3	<0.001
Esophagus	3.6±2.7	4.5±3.2	<0.001
Cervix	1.8±2.2	5.3±4.0	<0.001

Table 4 shows the results of stratificating all patients by the adjusted-ACCI risk score into four risk groups. The mean age of these four groups were 53±9, 61±12, 76±8, 81±6 years, respectively. Among patients who underwent surgical procedures for stomach, colorectal, prostate, or pancreas cancer, more than 20 percent were in the risk groups 8–11 and ≧12.

**Table 4:** Baseline characteristics of cancer surgery patients, *n* = 156,151.

Variable	Total	AACCI score 0–3	AACCI score 4–7	AACCI score 8–11	AACCI score≧12	*P*-value
	*n*(%)	*n*(%)	*n*(%)	*n*(%)	*n*(%)	
Total	156151	79227(50.7)	53326(34.2)	18877(12.1)	4721(3.0)	
Mean age, years ±SD	60±13	53±9	61±12	76±8	81±6	<0.001
Gender						<0.001
Female	77519	43084(55.6)	25287(32.6)	7317(9.4)	1831(2.4)	
Male	78632	36143(46.0)	28039(35.7)	11560(14.7)	2890(3.7)	
Cancer type						<0.001
Lung and bronchus	7101	3334(47.0)	2779(39.1)	866(12.2)	122(1.7)	
Liver and intrahepatic bile duct	13252	6796(51.3)	4533(34.2)	1590(12.0)	333(2.5)	
Colorectum	44737	15245(34.1)	17890(40.0)	8899(19.9)	2703(6.0)	
Breast	40569	26903(66.3)	11793(29.1)	1631(4.0)	242(0.6)	
Oral cavity and pharynx	24066	15752(65.5)	6796(28.2)	1351(5.6)	167(0.7)	
Stomach	10396	3287(31.6)	4005(38.5)	2355(22.7)	749(7.2)	
Prostate	7940	2940(37.0)	3056(38.5)	1643(20.7)	301(3.8)	
Pancreas	1375	475(34.5)	604(43.9)	232(16.9)	64(4.7)	
Esophagus	2612	1271(48.7)	1106(42.3)	204(7.8)	31(1.2)	
Cervix	4103	32247(78.6)	764(18.6)	106(2.6)	9(0.2)	
With metastatic disease	38254	0(0.0)	26485(69.2)	8324(21.8)	3445(9.0)	<0.001
Hospital characteristics						
Teaching level						<0.001
Medical center	99375	50817(51.1)	34229(34.4)	11471(11.5)	2858(2.9)	
Regional	50616	25281(49.9)	17244(34.1)	6449(12.7)	1642(3.2)	
District	6160	3129(50.8)	1853(30.1)	957(15.5)	221(3.6)	

Abbreviations: AACCI score, adjusted age-adjusted Charlson comorbidity index score.

**Fig 1:**
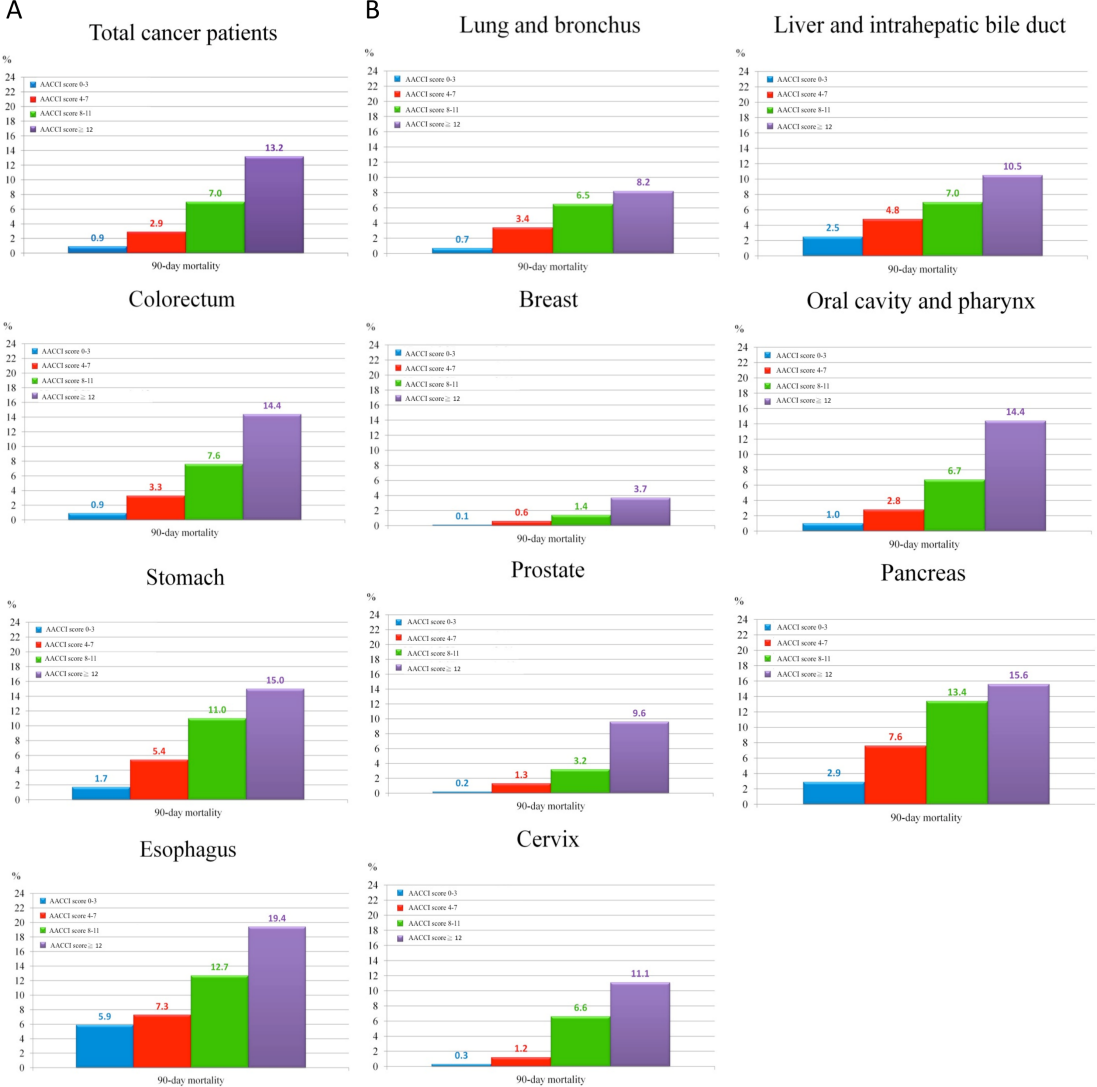
(A) 90-day mortality rate for the overall cohort by adjusted-ACCI score. (B) 90-day mortality rate for individual caner type by adjusted-ACCI score.

Table 5 and Fig 1 showed that stratificating patients by adjusted-ACCI score into four risk groups resulted in a gradient for mortality estimates. The 90-day mortality rates for the overall cohort was 2.7% and for each group was 0.9%, 2.9%, 7.0%, and 13.2%, respectively. The estimated 90-day mortality rates of each cancer surgery after stratification by the risk score are also demonstrated in Table 4 and Fig 1.

**Table 5:** Mortality of cancer surgery patients by cancer types and adjusted age-adjusted Charlson Comorbidity Index Score from 2007 to 2011, *n* = 156,151.

Variables	Event (%)	*P*-value
Total cancer patients (*n* = 156151)	4198(2.7)	<0.001
AACCI score 0–3 (*n* = 79227)	684(0.9)	
4–7 (*n* = 53326)	1559(2.9)	
8–11 (*n* = 18877)	1330(7.0)	
≧12 (*n* = 4721)	625(13.2)	
Lung and bronchus (*n* = 7101)	185(2.5)	<0.001
AACCI score 0–3 (*n* = 3334)	25(0.7)	
4–7 (*n* = 2779)	94(3.4)	
8–11 (*n* = 866)	56(6.5)	
≧12 (*n* = 122)	10(8.2)	
Liver and intrahepatic bile duct (*n* = 13252)	532(4.0)	<0.001
AACCI score 0–3 (*n* = 6796)	169(2.5)	
4–7 (*n* = 4533)	216(4.8)	
8–11 (*n* = 1590)	112(7.0)	
≧12 (*n* = 333)	35(10.5)	
Colorectum (*n* = 44737)	1802(4.0)	<0.001
AACCI score 0–3 (*n* = 15245)	143(0.9)	
4–7 (*n* = 17890)	598(3.3)	
8–11 (*n* = 8899)	672(7.6)	
≧12 (*n* = 2703)	389(14.4)	
Breast (*n* = 40569)	131(0.3)	<0.001
AACCI score 0–3 (*n* = 26903)	26(0.1)	
4–7 (*n* = 11793)	73(0.6)	
8–11 (*n* = 1631)	23(1.4)	
≧12 (*n* = 242)	9(3.7)	
Oral cavity and pharynx (*n* = 24 066)	463(1.9)	<0.001
AACCI score 0–3 (*n* = 15752)	161(1.0)	
4–7 (*n* = 6796)	187(2.8)	
8–11 (*n* = 1351)	91(6.7)	
≧12 (*n* = 167)	24(14.4)	
Stomach (*n* = 10396)	642(6.2)	<0.001
AACCI score 0–3 (*n* = 3287)	55(1.7)	
4–7 (*n* = 4005)	216(5.4)	
8–11 (*n* = 2355)	259(11.0)	
≧12 (*n* = 749)	112(15.0)	
Prostate (*n* = 7940)	128(1.6)	<0.001
AACCI score 0–3 (*n* = 2940)	7(0.2)	
4–7 (*n* = 3056)	39(1.3)	
8–11 (*n* = 1643)	53(3.2)	
≧12 (*n* = 301)	29(9.6)	
Pancreas (*n* = 1375)	101(7.3)	<0.001
AACCI score 0–3 (*n* = 475)	14(2.9)	
4–7 (*n* = 604)	46(7.6)	
8–11 (*n* = 232)	31(13.4)	
≧12 (*n* = 64)	10(15.6)	
Esophagus (*n* = 2612)	188(7.2)	<0.001
AACCI score 0–3 (*n* = 1271)	75(5.9)	
4–7 (*n* = 1106)	81(7.3)	
8–11 (*n* = 204)	26(12.7)	
≧12 (*n* = 31)	6(19.4)	
Cervix (*n* = 4103)	26(0.6)	<0.001
AACCI score 0–3 (*n* = 3224)	9(0.3)	
4–7 (*n* = 764)	9(1.2)	
8–11 (*n* = 106)	7(6.6)	
≧12 (*n* = 9)	1(11.1)	

Abbreviations: AACCI score, adjusted age-adjusted Charlson comorbidity index score.

Table 6 shows the results of the multivariate logistic regression analysis. After adjusting for these variables, the adjusted-ACCI score was strongly associated with 90-day mortality risk. The adjusted hazard ratio of the three higher score groups were 2.84 (95% CI, 2.59–3.12; *P* < 0.001), 6.07 (95% CI, 5.51–6.68; *P* < 0.001), and 11.17 (95% CI, 9.97–12.50; *P* < 0.001), respectively, in regard to 90-day mortality compared to the lowest score group. Operations for esophageal and pancreas cancers had higher HR of 15.80 (95% CI, 12.51–19.95; *P* < 0.001) and 13.06 (95% CI, 10.02–17.02; *P* < 0.001), respectively, than HR for breast cancer surgery. Males had a higher risk of perioperative mortality than did females (*P* < 0.001). Patients treated at regional and district hospitals had a higher risk of perioperative mortality than those treated at medical centers (*P* < 0.001).

**Table 6:** Multivariate logistic regression analysis.

Variable	Adjusted HR*	95% CI	*P*-value
AACCI score			
0–3	1		
4–7	2.84	2.59–3.12	<0.001
8–11	6.07	5.51–6.68	<0.001
≧12	11.17	9.97–12.50	<0.001
Cancer type			
Breast	1		
Lung and bronchus	8.49	6.96–10.36	<0.001
Liver and intrahepatic bile duct	5.62	4.47–7.07	<0.001
Colorectum	6.02	5.01–7.25	<0.001
Oral cavity and pharynx	5.13	4.18–6.30	<0.001
Stomach	8.84	7.26–10.76	<0.001
Prostate	2.29	1.77–2.96	<0.001
Pancreas	13.06	10.02–17.02	<0.001
Esophagus	15.80	12.51–19.95	<0.001
Cervix	2.47	1.62–3.76	<0.001
Gender			
Female	1		
Male	1.22	1.14–1.31	<0.001
Hospital characteristics			
Medical center	1		
Regional	1.43	1.34–1.53	<0.001
District	2.19	1.93–2.48	<0.001

Abbreviations: AACCI, adjusted age-adjusted Charlson comorbidity index; HR, hazard ratio; 95% CI, 95% confidence interval.

*Adjust for the patients’ adjusted age-adjusted Charlson comorbidity index score, gender, cancer type, and hospital characteristics.

Table 7 shows the distribution of the adjusted HR for 90-day mortality by cancer subtypes with the adjusted-ACCI risk score. Almost in all of the cancer surgeries, patients with scores of 4–7, 8–11 and ≧12 had significantly higher mortality risks compared to those with scores of 0–3. The postoperative 90-day mortality risk increased as the risk score increased was consistent in all cancers.

**Table 7:** Hazard ratios of individual adjusted age-adjusted Charlson comorbidity index score for mortality.

Variable	Adjusted HR*	95% CI
Total cancer patients		
AACCI score 0–3	1	
4–7	2.84	2.59–3.12
8–11	6.07	5.51–6.68
≧12	11.17	9.97–12.50
Lung and bronchus		
AACCI score 0–3	1	
4–7	4.07	2.61–6.33
8–11	7.42	4.61–11.92
≧12	8.66	4.14–18.10
Liver and intrahepatic bile duct		
AACCI score 0–3	1	
4–7	1.91	1.56–2.34
8–11	2.83	2.23–3.59
≧12	4.31	2.99–6.21
Colorectum		
AACCI score 0–3	1	
4–7	3.63	3.03–4.36
8–11	8.29	6.92–9.94
≧12	16.40	13.54–19.87
Breast		
AACCI score 0–3	1	
4–7	6.74	4.30–10.56
8–11	14.64	8.35–25.65
≧12	37.05	17.35–79.09
Oral cavity and pharynx		
AACCI score 0–3	1	
4–7	2.71	2.20–3.35
8–11	6.77	5.23–8.76
≧12	15.38	10.02–23.62
Stomach		
AACCI score 0–3	1	
4–7	3.21	2.39–4.32
8–11	6.62	4.95–8.87
≧12	9.53	6.90–13.17
Prostate		
AACCI score 0–3	1	
4–7	5.14	2.29–11.50
8–11	12.73	5.77–28.08
≧12	41.48	18.15–94.81
Pancreas		
AACCI score 0–3	1	
4–7	2.79	1.53–5.10
8–11	4.87	2.58–9.18
≧12	5.67	2.51–12.80
Esophagus		
AACCI score 0–3	1	
4–7	1.24	0.91–1.70
8–11	2.30	1.47–3.60
≧12	3.84	1.66–8.84
Cervix		
AACCI score 0–3	1	
4–7	4.29	1.70–10.83
8–11	23.80	8.83–64.13
≧12	43.69	5.51–346.49

*Adjust for the patients’ adjusted age-adjusted Charlson comorbidity index score, gender, cancer type, and hospital characteristics.

Abbreviations: AACCI, adjusted age-adjusted Charlson Comorbidity Index; Adjusted HR, Adjusted hazard ratio; 95% CI, 95% confidence interval.

## Discussion

In this study from a national database identifying 156,151 cancer operations between 2007 and 2011 in Taiwan, we developed and validated an adjusted-ACCI model to predict the risk of perioperative mortality after cancer surgery. The adjusted-ACCI model for predicting 90-day mortality after cancer surgery was different from the original ACCI model that they weighted differently in regard to comorbidity variables. Variables that were strongly associated with 90-day mortality risk after cancer surgery in the adjusted-ACCI were for those over 80 years of age, 70–80 years of age as well as those with pre-existing renal disease. The overall perioperative mortality for common cancer types leading to death in Taiwan between 2007 and 2011 was 2.7%, while the mortality rate according to risk score groups ranged from 0.9% to 13.2%. When the data were stratified by risk score, cancer patients with higher scores were at a greater risk of perioperative mortality. The adjusted-ACCI score provided a quantification of the magnitude of the prognostic impact of age and comorbidity for the perioperative mortality in cancer surgery.

The strength of this study is that it is a nationwide population-based study that included almost all the patients who had cancer surgery for the common leading cancer types for deaths in Taiwan. By the end of 2006, the NHI covered 99.0% of Taiwan’s population, with nearly complete follow-up information of mortality among the entire study population, and the NHI Bureau of Taiwan routinely monitored the dataset for diagnostic accuracy. The results of this population-based study are noteworthy since perioperative death from cancer surgery is rare so that individual studies are generally too small to detect measureable differences, and mortality associated with inpatient surgery varies among various hospitals [28,29].

Quantification of acceptable risk for individuals is important since a surgical procedure is always associated with potential benefits that need to be weighed against potential risks when deciding whether to perform an operation. With the advance in management of cancer diseases and surgical techniques, the perioperative mortality rate has declined over the last few decades [4]. Even for digestive system cancer operations, our results show that the overall 90-day mortality rates were no more than 7.3% over the five years studied. Although inherent operation complexity is one of the determinants of perioperative mortality, it has been predicted that when difficult operations become standardized and operative mortality falls, the patient’s generic risk factors may become the major contributors to operative mortality [30].

More and more of the elderly population are undergoing surgery, therefore, in our adjusted-ACCI model we took the parameter of over 80 years of age as an independent variable, which was previously included in the variable of over the age of 70 in the original ACCI, into analysis. Our results indicate that those aged over 80 were strongly at risk for short-term mortality in cancer surgery, which was in line with some previous studies [31–33]. Although old age is strongly associated with short-term mortality, it has been suggested that age alone is not a contraindication to surgery in certain cancer patients [34,35]. Some single-institution or multicenter studies have shown that in certain operations patients older than 80 had equivalent mortality rates with younger patients [35–37]. Despite greater morbidity, the benefit of surgery did not diminish with old age in selected cancer patients [35,38]. The results from these studies indicated the importance of identifying patients with higher risks of mortality among the elderly population before surgery. The present study suggests that the adjusted-ACCI score could be used to account for the impact of age and comorbidities on mortality in cancer surgery. Patients over 80 years of age have a higher risk of perioperative mortality, but they may tolerate the operation well if they are otherwise healthy. However, if they have one of the comorbidities listed in the adjusted-ACCI, the mortality risk may increase largely. Those younger than 80 may be at even greater risk of perioperative mortality if they have more than one of the comorbidities listed in the adjusted-ACCI.

In this study, the average adjusted-AACI score of survival and mortality group varied from cancer to cancer. In general, when a patient of a certain cancer comes with a score higher than the average, his perioperative mortality rate would be higher than the average for this type of cancer. The average adjusted-AACI score may be considered as one of the cut-off points that warns surgeons that the mortality rate might be higher than the average. In some cases, surgeons may have multiple cut-off points representing tiered levels of risk for cancer patients. Meanwhile, the present study provides four risk score groups with estimates of mortality rate and mortality risk which may help surgeons to determine whether the surgery is worth the risk or not. When treating certain cancer patients with a high adjusted-ACCI score, the estimates from the score should be discussed with the patient during preoperative assessment and counseling. In addition, meticulous surgical techniques and perioperative management are crucial for the high-risk group to lower the mortality risk.

In preoperative risk measure, the American Society of Anesthesiology’s (ASA) physical status classification is currently the most widely used and standard component of preoperative assessment for surgical patients, however it does not incorporate age. In the ASA classification the majority of patients, young or old, with comorbidities are classified as ASA class 2 and 3. Patients over 80 years of age with mild systemic disease will be classified the same as younger patients with mild systemic disease. In this case, the elderly patient’s perioperative mortality risk might be underestimated. The present study tries to weigh the impact of age as well as each of the other major comorbidities on perioperative mortality risk after cancer surgery. In addition to the widely used ASA classification, the score with risk estimates in this study might be helpful for cancer patient counseling and informed consent.

There are some limitations in this study. First, patients’ diagnoses and identification of comorbidities were completely dependent on ICD codes, and any coding errors in patients’ underlying diseases could lead to disparities in the recorded numbers of pre-existing comorbidities. Nonetheless, the NHI Bureau of Taiwan randomly reviews the charts and interviews patients in order to verify diagnostic accuracy [39]. Second, we used administrative data, which was limited in the ability to account fully for the severity of comorbidities. Third, the ACCI variable was used to analyze the risk of perioperative mortality, but it is possible that other factors not included in the ACCI could be contributing to perioperative mortality. Fourth, rather than surgical mortality, all-cause mortality was used. However, the short-term surgical and all-cause mortality differed only slightly. Finally, the cancer stage was not included in the NHIRD database; therefore we were unable to further analyze the impact of different stages of each cancer on perioperative mortality risk in this study. In the literature, some studies from single-institutions or high-volume centers’ reports showed that cancer stage was not correlated with perioperative mortality in some cancers[40–42]. But the risk point of the metastatic disease (stage 4) in this study weighed 4 points, which was a considerable factor. It might give us the idea that advanced cancer stage (stage 4) might affect the perioperative mortality to some degree. Further studies with cancer stage might be needed to reveal a deeper insight about the different stages of cancer and their relationship with perioperative mortality after surgery. Despite these limitations, the adjusted-ACCI measure allows integration of both age and comorbidities into clinical preoperative decision-making. This combination is more inclusive than either age or comorbidity alone. Moreover, the present study used a national database, including almost all major cancers associated with the greatest number of perioperative deaths, which increased its generalizability. Although there is a trade-off between generalizability and precision, and drawing these estimates from a national database may yield estimates with a degree of error in regard to individual cancer patients, the purpose of the adjusted-ACCI score is to provide a generalized risk estimate. The risk estimate may be more reliable than the estimate derived from a single-institution or from high-volume center reports.

## Conclusions

In summary, as surgical technologies improve and neo-adjuvant and adjuvant therapies advance, the number of advanced-age cancer patients, with their attendant comorbidities, who will be considered for surgical treatment will increase. Our results demonstrate that this adjusted-ACCI score could be used to identify patients with a higher risk of 90-day mortality after cancer surgery. In any individual case, that the risks of death related to cancer surgery vary for specific cancer types, but those over 80 years of age accompanied with any comorbidities of significant association with mortality might account for the majority of perioperative deaths. The adjusted-ACCI score may be useful for preoperative risk stratification, decision-making, and counseling of individual cancer patients, particularly for those over 80 years of age.
