# Correction: Adjusted Age-Adjusted Charlson Comorbidity Index Score as a Risk Measure of Perioperative Mortality before Cancer Surgery

**DOI:** 10.1371/journal.pone.0157900

**Published:** 2016-06-15

**Authors:** Chun-Ming Chang, Wen-Yao Yin, Chang-Kao Wei, Chin-Chia Wu, Yu-Chieh Su, Chia-Hui Yu, Ching-Chih Lee

The phrase “hazard ratios” appears incorrectly throughout the article. The correct phrase should be “odds ratios.”

In Table 2, the column headings “Adjusted HR” should be “Adjusted OR”. Please see the corrected Table 2 here.

**Table 2:** Development of the predictor score based on multivariate logistic regression analysis of the derivation set, n = 78,076.

	Coefficient β	Adjusted OR	95% CI	*P*-value	Risk score
50≧Age		1			
60≧Age>50	0.293	1.34	1.13–1.58	0.001	1
70≧Age>60	0.635	1.88	1.59–2.22	<0.001	3
80≧Age>70	1.289	3.63	3.11–4.23	<0.001	5
Age>80	2.129	8.40	7.16–9.86	<0.001	9
Congestive heart failure	0.722	2.05	1.53–2.76	<0.001	3
Dementia	0.763	2.14	1.21–3.78	0.008	3
Chronic pulmonary disease	0.319	1.37	1.11–1.69	0.003	1
Peptic Ulcer Disease	0.555	1.74	1.49–2.03	<0.001	2
Diabetes Mellitus	0.319	1.37	1.18–1.60	<0.001	1
Renal disease	1.131	3.09	2.34–4.10	<0.001	5
Lymphoma	1.000	2.71	1.43–5.13	0.002	4
Any malignancy	0.245	1.27	1.10–1.47	0.001	1
Metastatic solid tumor	0.918	2.50	2.28–2.74	<0.001	4
Mild Liver Disease	0.754	2.12	1.73–2.59	<0.001	3
Moderate Liver Disease	1.018	2.76	1.93–3.95	<0.001	4

Abbreviation: Adjusted OR, Adjusted odds ratio; 95%CI, 95% confidence interval.

In Table 6, the column headings “Adjusted HR” should be “Adjusted OR”. Please see the corrected Table 6 here.

**Table 6:** Multivariate logistic regression analysis.

Variable	Adjusted OR*	95% CI	*P*-value
AACCI score			
0–3	1		
4–7	2.84	2.59–3.12	<0.001
8–11	6.07	5.51–6.68	<0.001
≧12	11.17	9.97–12.50	<0.001
Cancer type			
Breast	1		
Lung and bronchus	8.49	6.96–10.36	<0.001
Liver and intrahepatic bile duct	5.62	4.47–7.07	<0.001
Colorectum	6.02	5.01–7.25	<0.001
Oral cavity and pharynx	5.13	4.18–6.30	<0.001
Stomach	8.84	7.26–10.76	<0.001
Prostate	2.29	1.77–2.96	<0.001
Pancreas	13.06	10.02–17.02	<0.001
Esophagus	15.80	12.51–19.95	<0.001
Cervix	2.47	1.62–3.76	<0.001
Gender			
Female	1		
Male	1.22	1.14–1.31	<0.001
Hospital characteristics			
Medical center	1		
Regional	1.43	1.34–1.53	<0.001
District	2.19	1.93–2.48	<0.001

Abbreviations: AACCI, adjusted age-adjusted Charlson comorbidity index; OR, odds ratio; 95% CI, 95% confidence interval.

*Adjust for the patients’ adjusted age-adjusted Charlson comorbidity index score, gender, cancer type, and hospital characteristics.
